# Association of sex and age on outcomes of maintenance treatment in pati​ents with advanced esophagogastric adenocarcinoma: an exploratory analysis of the randomized AIO-YMO-0111-MATEO trial

**DOI:** 10.1007/s00432-026-06662-y

**Published:** 2026-11-13

**Authors:** G. Stocker, R. Stelmach, S. Lorenzen, T. Ettrich, M. Knödler, F. Longo, B. Chibaudel, R. Greil, M. Perwög, J. Larcher-Steiner, S. Held, F. Lordick, G. M. Haag

**Affiliations:** 1 Department of Medicine II (Oncology, Gastroenterology, Hepatology and Pulmonology), University Cancer Center Leipzig, University of Leipzig Medical Center, Leipzig, Germany; 2 Department of Medical Oncology, National Center for Tumor Diseases, Heidelberg University Hospital, Heidelberg, Germany; 3 Clinic and Policlinic for Internal Medicine III, School of Medicine, Technical University of Munich, Munich, Germany; 4 Department of Internal Medicine I, University Hospital Ulm, Ulm, Germany; 5 Charité Comprehensive Cancer Center, Charité – Universitätsmedizin Berlin, Corporate Member of Freie Universität Berlin und Humboldt-Universität zu Berlin, Berlin, Germany; 6 Medical Department, Ramon y Cajal University Hospital, Madrid, Spain; 7 Department of Medical Oncology Department, HFB - Hoptial Franco-Britannique, Fondation Cognacq-Jay, Levallois-Perret, France; 8 3rd Medical Department, Paracelsus Medical University, Salzburg, Austria; 9 P95/Julius Clinical, Innsbruck, Austria; 10 AIO-Studien-gGmbH, Berlin, Germany; 11 Clinical Cooperation Unit Applied Tumor-Immunity, German Cancer Research Center (DKFZ), Heidelberg, Germany

**Keywords:** Esophagogastric adenocarcinoma, Sex differences, Age, Chemotherapy, Prognostic factors

## Abstract

**Purpose:**

Sex and age have an impact on efficacy and toxicity of chemotherapy (ctx) in patients (pts) with esophagogastric adenocarcinoma (EGA). However, data are limited, as clinical trials frequently do not report sex-specific differences and older pts are often underrepresented.

**Methods:**

MATEO is an international randomized trial, confirming non-inferiority of S-1 maintenance therapy compared to continued platinum-based ctx in pts with advanced EGA without disease progression after 3 months of ctx. Toxicity pattern favored S-1 monotherapy. We conducted a post hoc exploratory analysis to assess the impact of sex and age on treatment outcomes.

**Results:**

In the per-protocol population, 112 pts (72.7%) were male and 42 (27.3%) were female. 37.7% of pts were ≥ ​70 years. Female pts more often had distal stomach tumors (64.3%), while male pts had more esophagogastric junction tumors (76.7%). In a pooled analysis, female pts showed significantly longer progression-free survival (PFS) (median 9.9 vs. 4.3 months, HR 0.56 [95% CI 0.38–0.83], log-rank: *p* = 0.0033), and overall survival (OS) (median 14.4 vs. 12.2 months, HR 0.65 [95% CI 0.44–0.97], log-rank: *p* = 0.0348). No age-related differences in PFS and OS were observed between pts ≥ 70 years and < 70 years. In multivariate analyses, female sex, but not younger age, was associated with a trend towards improved OS. No significant sex- or age-specific differences in terms of toxicity were observed.

**Conclusion:**

Primary tumor location differed by sex. A trend for superior outcome in female pts was observed, whereas age did not have a significant impact on outcome.

**Supplementary Information:**

The online version contains supplementary material available at https://doi.org/10.1007/s00432-026-06662-y.

## Introduction

Esophagogastric adenocarcinoma (EGA), encompassing adenocarcinomas of the esophagus, esophagogastric junction, and stomach, represents a major global health burden with a global incidence of ~ 9.2/100.000 (Bray et al. 2024). Despite therapeutic improvements, prognosis of advanced, unresectable HER2-negative disease remains poor with a median overall survival (OS) of approximately 13 months with contemporary systemic treatment strategies (Rha et al. 2023; Janjigian et al. 2026). Platinum–fluoropyrimidine-based ctx regimens with or without immunotherapeutic or targeted agents represent the backbone of first-line treatment; however these are associated with significant short-term and long-term toxicity.

Sex-based differences in cancer biology, pharmacokinetics, and treatment outcomes have gained increasing attention in oncology. In EGA, males are disproportionately affected, with male-to-female ratios exceeding 4:1 for esophageal adenocarcinoma and approximately 2:1 for gastric adenocarcinoma (Davidson et al. 2019; Athauda et al. 2020; Dijksterhuis et al. 2021; Plazas et al. 2022). This imbalance has led to a relative underrepresentation of female pts in clinical trials, limiting the ability to detect sex-specific differences in treatment efficacy and toxicity. In the advanced disease setting of EGA, higher rates of nausea, vomiting, diarrhea, stomatitis, and alopecia, as well as more frequent serious adverse events (AEs), have been reported in female pts in a pooled analysis of 1,654 pts. No significant sex differences were observed in univariate analyses for progression-free survival (PFS) or OS. In contrast, multivariate analysis suggested a potential survival advantage for female pts with respect to both PFS and OS (Davidson et al. 2019; Plazas et al. 2022).

In addition, age has a strong impact on treatment tolerance and might require adaptation of therapeutical regimens. Younger pts (< 65 years) with a high need for rapid disease control and unfavorable histology (non-intestinal subtype) may benefit from intensification of treatment by adding docetaxel (Zaanan et al. 2025), although triplet regimens are associated with increased toxicity (Yamada et al. 2019; Zaanan et al. 2025). On the other hand, in older pts, often presenting with a reduced general condition reflecting frailty, reduced ctx intensity has demonstrated efficacy with a more favorable toxicity pattern (Hall et al. 2021).

The international randomized phase II MATEO trial evaluated S1-maintenance following a 3-month induction phase (consisting of a platinum–fluoropyrimidine doublet or triplet-regimen) demonstrated non-inferiority of S-1 maintenance therapy compared with continued platinum-based combination ctx in pts with advanced HER2-negative EGA, with a significantly lower incidence of grade ≥ 2 peripheral sensory polyneuropathy in the S-1 maintenance arm (Stocker et al. 2023).

The present post hoc analysis aims to investigate the impact of sex and age on treatment outcomes within the MATEO trial population.

## Methods

### Study design and patients

The design and primary results of the MATEO trial have been published previously (Haag et al. 2017; Stocker et al. 2023). Briefly, MATEO was an international, randomized, open-label phase II trial conducted across multiple European centers. Pts with histologically confirmed HER2-negative advanced EGA without progression or limiting toxicity after 12 weeks of first-line platinum-fluoropyrimidine-based induction ctx were randomized in a 2:1 allocation to receive S-1 monotherapy (arm A) or to continue combination ctx (arm B).

The primary endpoint of the trial was OS, with a non-inferiority design. Secondary endpoints included PFS, AEs, and quality of life (Stocker et al. 2023). Recently, the impact of nutritional risk screening (NRS) on outcomes in pts undergoing induction therapy within the MATEO population has been reported (Stelmach et al. 2026).

### Statistical analysis

Following the principles of the initial non-inferiority design, efficacy analyses were conducted in the per-protocol population (including all randomized pts who have started maintenance treatment without significant protocol violations), safety analyses were conducted in the safety population (including all randomized pts who have started maintenance treatment). Pts were stratified by sex (male vs. female) and age (< 70 vs. ≥70 years). Baseline characteristics, including primary tumor location, were compared between subgroups. Since the primary analysis demonstrated non-inferiority between arm A and arm B and this post-hoc analysis aimed to evaluate the prognostic impact of sex and age, survival outcomes were analyzed in a pooled analysis of both treatment arms according to sex and age. PFS and OS since randomization (after 3 month of induction ctx) were estimated using the Kaplan-Meier method and compared using the log-rank test. Hazard ratios (HRs) and corresponding 95% confidence intervals (CIs) were estimated using Cox proportional hazard regression models. AEs were compared between subgroups using descriptive statistics, categorical variables were compared using two-sided Fisher’s exact tests. A two-sided significance level of 5% was applied; accordingly, p-values < 0.05 were considered statistically significant. Multivariate cox regression models included sex, age, primary tumor location, and response to induction ctx as covariates with or without adjusting for treatment arm.

## Results

### Patient characteristics

The per-protocol population consisted of 154 pts: Of these, 112 (72.7%) were male and 42 (27.3%) were female. The median age was 65 years in both sex groups. However, a higher proportion of female pts were aged 70 years or older (45.2% vs. 34.8%). Female pts had a higher rate of distal stomach tumors (64.3%), while a predominance of esophagogastric junction tumors (76.7%) was observed in male pts. Male sex was associated with a slightly better Eastern Cooperative Oncology Group Performance Status (ECOG PS) compared to female sex, with a proportion of ECOG PS 0 of 58.0% vs. 45.2%. Regarding induction ctx, male pts more frequently received the triplet regimen FLOT (50.0% vs. 35.7%).

Overall, 96 pts (62.3%) were aged younger than 70 years, whereas 58 (37.7%) were aged ≥ 70 years. Compared with younger pts, those aged ≥ 70 years more frequently had an ECOG PS of 1 (56.9% vs. 38.5%) and received FLOT induction ctx (15.5% vs. 64.6%) less frequently.

Detailed patient characteristics stratified by sex and age group are summarized in Table 1.

**Table 1:** Patient characteristics according to sex and age

	Male patients *n* = 112	Female patients *n* = 42	< 70 years *n* = 96	≥ 70 years *n* = 58
Age				
median [years]	65	65		
min – max [years]	35–84	34–81		
< 70 years, *n* [%]	73 (65.2)	23 (54.8)		
≥ 70 years, *n* [%]	39 (34.8)	19 (45.2)		
Sex				
Male, *n* [%]			73 (76.0)	39 (67.2)
Female, *n* [%]			23 (24.0)	19 (32.8)
ECOG PS				
0, *n* [%]	65 (58.0)	19 (45.2)	59 (61.5)	25 (43.1)
1, *n* [%]	47 (42.0)	23 (54.8)	37 (38.5)	33 (56.9)
Treatment Arm				
A (S-1), *n* [%]	72 (64.3)	33 (78.6)	68 (70.8)	37 (63.8)
B (cont. poly ctx regimen), *n* [%]	40 (35.7)	9 (21.4)	28 (29.2)	21 (36.2)
Localisation				
Distal esophagus (AEG I), *n* [%]	36 (32.1)	6 (14.3)	28 (29.2)	14 (24.1)
Cardia carcinoma (AEG II), *n* [%]	37 (33.0)	5 (11.9)	26 (27.1)	16 (27.6)
Subcardial carcinoma (AEG III), *n* [%]	13 (11.6)	4 (9.5)	7 (7.3)	10 (17.2)
Distal stomach	26 (23.2)	27 (64.3)	35 (36.5)	18 (31.0)
Histological subtype				
Intestinal type, *n* [%]	46 (41.4)	17 (40.5)	40 (42.1)	23 (39.7)
Diffuse type, *n* [%]	20 (18.0)	11 (26.2)	20 (21.1)	11 (19.0)
Mixed type, *n* [%]	10 (9.0)	4 (9.5)	9 (9.5)	5 (8.6)
Unknown, *n* [%]	36 (32.1)	10 (23.8)	26 (27.4)	19 (32.8)
Prior chemotherapy, *n* [%]	19 (17)	7 (16.7)	18 (18.8)	8 (13.8)
Prior radiotherapy, *n* [%]	8 (7.1)	2 ( 4.8)	7 (7.3)	3 (5.2)
Induction regimen				
FLOT, *n* [%]	56 (50.0)	15 (35.7)	62 (64.6)	9 (15.5)
FLO/mFOLFOX6, *n* [%]	45 (40.2)	27 (64.3)	26 (27.1)	46 (79.3)
Cisplatin-based doublet ctx, *n* [%] (Cisplatin/S-1, Cisplatin/5-FU and XP)	11 (9.8)	0	8 (8.3)	3 (5.1)

*ECOG PS *Eastern Cooperatve Oncology Group Performance Status, *cont*. continuous, *ctx *chemotherapy, *FLOT *docetaxel, oxaliplatin, 5-Fluorouracil and folic acid, FLO and mFOLFOX6, oxaliplatin, 5-fluorouracil and folic acid, S-1, tegafur, gimeracil and oteracil, *EOX/EOF *epirubicin, oxaliplatin and capcitabine or 5-fluorouracil, *XP *cisplatin and capecitabine

### Survival outcomes by sex

**Fig. 1:**
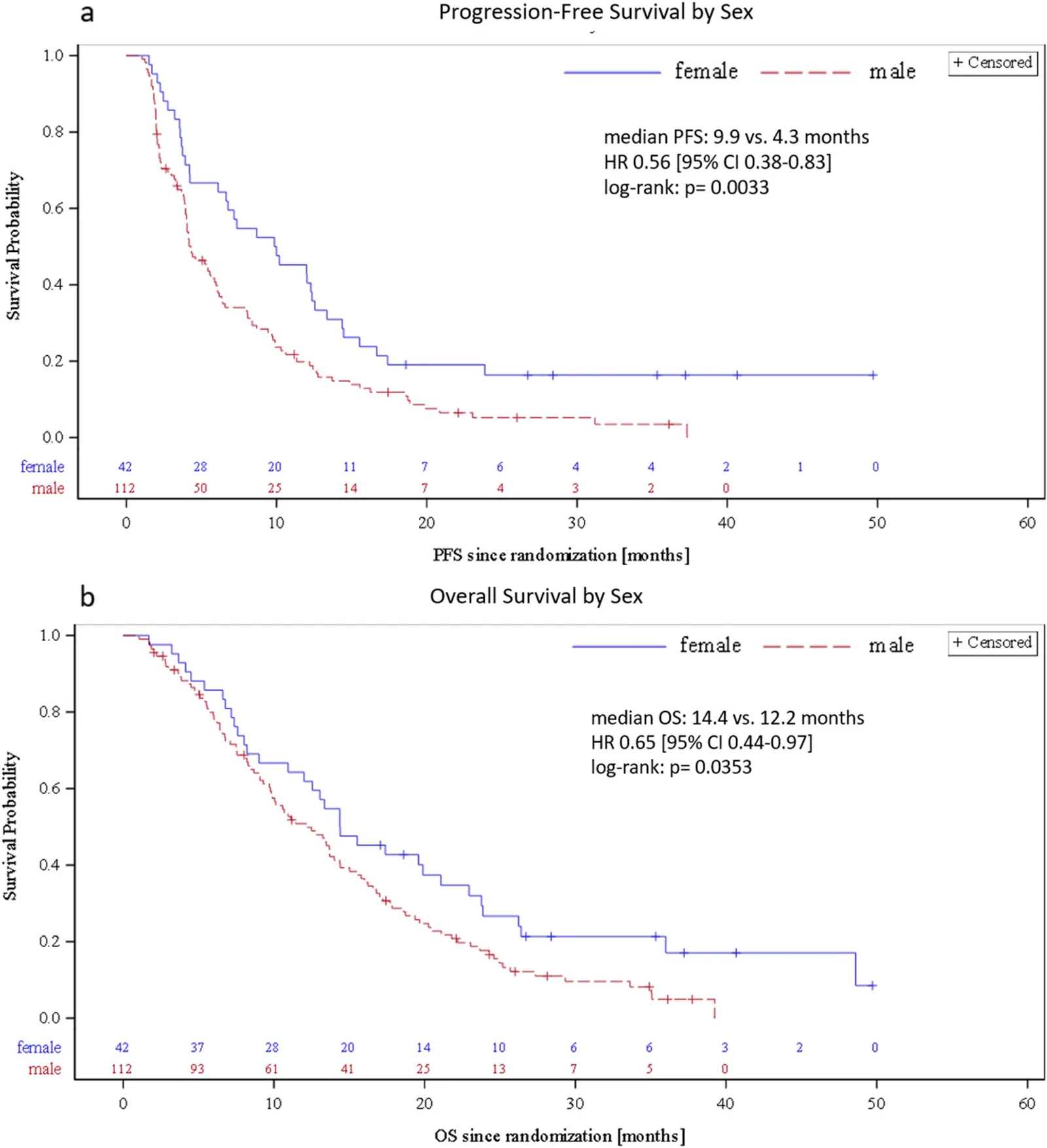
Kaplan-Meier estimates for **a** Progression-Free - and **b** Overall Survival by Sex since randomisation; Footnotes: HR, Hazard Ratio; CI, Confidence Interval

In the pooled analysis of arms A and B, female pts demonstrated a significantly longer PFS compared to male pts with a median of 9.9 months [95% CI 6.1–12.6] versus 4.3 months [95% CI 3.9–5.9] (HR 0.56 [95% CI 0.38–0.83], *p* = 0.0033). Similarly, OS was significantly superior in female pts with a median OS of 14.4 months [95% CI 10.9–21.1] compared to 12.2 months [95% CI 9.8–14.4] in male pts (HR 0.65 [95% CI 0.44–0.97], log-rank: *p* = 0.0348). The corresponding Kaplan-Meier estimates for PFS and OS by sex are shown in Fig. 1.

### Survival outcomes by age

**Fig. 2:**
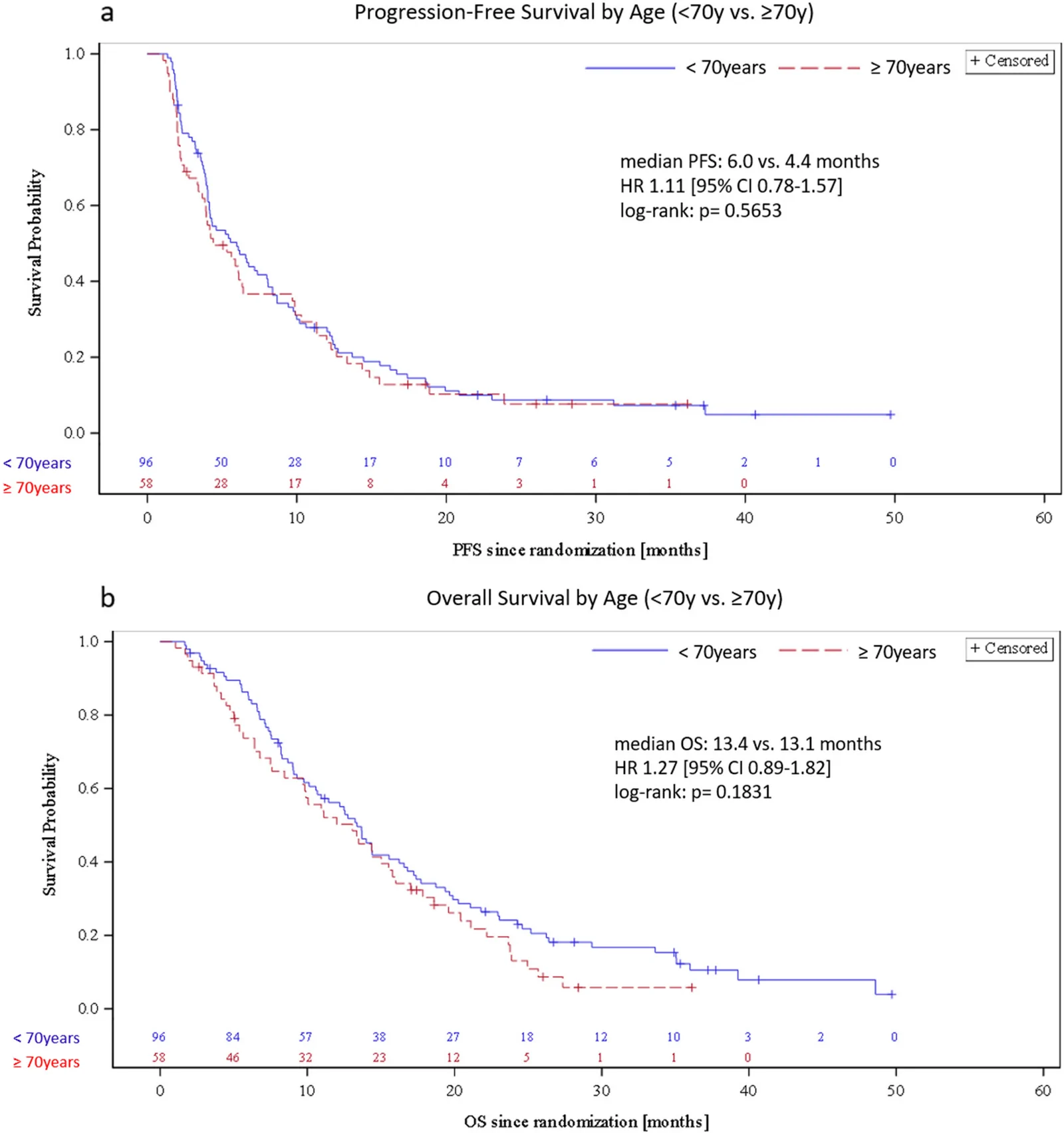
Kaplan-Meier estimates for **a** Progression-Free – and **b** Overall Survival by Age since randomisation; Footnotes: y, years; HR, Hazard Ratio; CI, Confidence Interval

No clinically meaningful age-related differences in PFS and OS were observed between younger and older pts. Median PFS in pts aged ≥ 70 years was 4.4 months [95% CI 3.7–6.4] vs. 6.0 months [95% CI 4.1–8.1] in pts aged < 70 years (HR 1.11 [95% CI 0.78–1.57], log-rank: *p* = 0.5653). Median OS was 13.1 months [95% CI 9.7–15.8] in pts aged ≥ 70 years vs. 13.4 months [95% CI 10.6–16.2] in pts aged < 70 years (HR 1.27 [95% CI 0.89–1.82], log-rank: *p* = 0.1831). The corresponding Kaplan-Meier estimates for PFS and OS by age are shown in Fig. 2.

### Multivariate analysis

In a multivariate Cox regression model including age, sex, primary tumor location, and response to induction ctx, response to induction therapy was associated with a prolonged OS as expected; in addition, a positive trend for OS in female pts was observed. These results were consistent, when the treatment arm was added to the multivariate analysis as an additional cofactor (Table 2).

**Table 2:** Multivariate cox regression models

Factor	Overall survival *n* = 154		
	HR	95% CI	*P*-value
Age			
< 70 years	1		0.1787
≥ 70 years	1.28	0.89–1.83	
Sex			
Male	1		0.0598
Female	0.66	0.43–1.02	
Primary tumor location			
Distal stomach	1		0.8173
AEG I-III	0.96	0.65–1.42	
Response to induction ctx			
SD/ Non—CR/ Non-PD	1		0.0465
CR/ PR	0.70	0.50–0.99	

Factor	Overall survival *n* = 154		
	HR	95% CI	*P*-value
Age			
< 70 years	1		0.1823
≥ 70 years	1.28	0.89–1.83	
Sex			
Male	1		0.0601
Female	0.66	0.43–1.02	
Primary tumor location			
Distal stomach	1		0.8177
AEG I-III	0.96	0.65–1.41	
Response to induction ctx			
SD/ Non—CR/ Non-PD	1		0.0466
CR/ PR	0.70	0.50–0.99	
Treatment arm			
Arm A (S-1)	1		0.9919
Arm B (combination ctx)	1.0	0.69–1.45	

*HR *hazard ratio, *CI *confidence interval, *AEG *adenocarcinoma of the esophagogastric junction, *ctx *chemotherapy, *SD *stable disease, *CR *complete remission, *PD *progressive disease

### Toxicity

In the safety population (155 pts), no clinically relevant difference in treatment-related AEs, severe AEs or serious AEs were observed between male and female pts or between < 70 and ≥ 70 years (Table 3).

**Table 3:** Treatment-related adverse events after randomisation

Safety population *n*= 155	Male patients *n*= 113 (%)	Female patients *n*= 42 (%)	*p*-value	<70y *n*= 96 (%)	≥70y *n*= 59 (%)	*p*-value
Any treatment-related AE	**99 (87.6)**	**37 (88.1)**	>0.9999	**81 (84.4)**	**55 (93.2)**	0.1324
Any treatment-related SAE	**8 (7.1)**	**1 (2.4)**	0.4457	**5 (5.2)**	**4 (6.8)**	0.7317
Any treatment-related AE grade ≥ CTC AE 3°	**32 (28.3)**	**11 (26.2)**	0.8428	**27 (28.1)**	**16 (27.1)**	>0.9999

*CTC *common toxicity criteria, *AE *adverse event, *SAE *serious adverse event

Toxicity was low as expected in a maintenance setting with a low incidences of grade 3 toxicities and rare grade 4 events. Regarding grade 1 and 2 toxicities, a heterogeneous pattern was observed between pts < 70 and ≥ 70 years as well as between male and female pts (supplementary Table 1). However, it should be taken into account that the ctx regimens administered differed between male and female pts, in addition more female pts received S-1 based maintenance therapy (78.6% vs. 64.3%) as randomization was not stratified by sex (Table 1).

## Discussion

This post hoc analysis of the MATEO trial provides important insights into the impact of sex and age on treatment outcomes in pts with advanced HER2-negative EGA, who completed 3 months of platinum-based ctx without disease progression and subsequently received either S-1 maintenance or continued combination therapy.

In accordance with other published studies, female pts presented with more distal stomach tumors, whereas primary tumors of the esophagogastric junction occurred predominantly in male. Tumor location appears to be of limited prognostic relevance in the metastatic setting, where biological tumor characteristics are likely to exert a greater influence on outcome than anatomical site (Chau et al. 2009). Whether female sex represents an independent positive prognostic factor in EGA remains inconsistently reported in retrospective studies, despite, in some cases, less favorable tumor biology (Davidson et al. 2019; Athauda et al. 2020; Plazas et al. 2022). In this analysis, female sex emerges as a prognostic factor in the univariate analysis and was associated with a favorable trend in the multivariate analyses, despite the fact that female pts were more commonly 70 years or older, and had more frequently a higher ECOG PS compared to male pts. It may be hypothesized that female pts have increased sensitivity to fluoropyrimidines, which becomes particularly evident in this study in the context of maintenance therapy. Evidence from earlier studies has long indicated sex-related differences in the pharmacokinetics of fluoropyrimidines; however, these findings have not been translated into sex-specific dosing recommendations (Milano et al. 1992; Yamashita et al. 2002; Mueller et al. 2013; Brooks et al. 2025). In addition, established differences in body composition between male and female sex are not sufficiently captured by body surface area–based dosing approaches for fluoropyrimidines (Lee et al. 2016; Drami et al. 2021).

Regarding toxicity, female pts with EGA or other malignancies, particularly those treated with fluoropyrimidines and oxaliplatin for colorectal cancer, have been reported to experience higher rates of treatment-related toxicity. This observation has been consistently demonstrated across multiple studies (Davidson et al. 2019; Athauda et al. 2020; Wagner et al. 2021, 2026; Plazas et al. 2022). In contrast, this association was not observed in the present analysis. However, it should be noted that toxicity was reported for the maintenance period, during which approximately two-thirds of pts received a de-escalated maintenance therapy after 3 months of induction treatment, and further dose reductions were permitted in the control arm as clinically indicated (44.9% in arm B; (Stocker et al. 2023)). It is therefore possible that the overall lower treatment intensity reflecting the maintenance phase contributed to the absence of increased toxicity in female pts in this cohort.

Age represents an important factor in the decision-making process regarding ctx intensity in first-line treatment of EGA. In the present analysis, age had no significant impact on outcome. Importantly, the MATEO trial included only pts with an ECOG PS of 0–1, and excluded pts who experienced disease progression during the 3-month induction ctx, thereby selecting a relatively fit population. In meta-analyses including randomized controlled trials reflecting a comparable study population, age was not identified as a relevant prognostic factor (Davidson et al. 2019; Athauda et al. 2020). There is a growing body of evidence suggesting that functional status and physiological reserve, rather than chronological age, are the primary determinants of treatment outcomes in older pts with EGA (Visa et al. 2018; Hayashi et al. 2019; Möhring et al. 2023).

Since the design and conduct of the MATEO study, the therapeutic landscape has undergone significant changes. In particular, ctx in combination with targeted therapies or in combination with checkpoint inhibitors has become standard of care as first-line treatment for most pts with EGA (Lordick et al. 2022, 2024). The efficacy of checkpoint inhibitors in the metastatic setting of EGA appears to correlate with the level of PD-L1 expression (Lee et al. 2025). A recent meta-analysis of immunotherapy trials in advanced gastroesophageal cancers demonstrated that both sexes with EGA benefit from immune checkpoint inhibitor-based therapy, with no significant interaction between sex and treatment effect (Masetti et al. 2026), whereas in the JAVELIN 100 trial, comparing avelumab maintenance therapy with continued ctx, subgroup analysis showed no benefit with checkpoint inhibitor-based maintenance therapy compared to ctx in female pts (Moehler et al. 2021). In addition, current evidence from meta-analyses indicates that the efficacy of checkpoint inhibitors is not significantly influenced by age in pts aged ≥ 65 years. However, data in very elderly populations (> 75 years) remain limited (Elias et al. 2018).

Several limitations should be acknowledged. First, this is a post hoc analysis with limited subgroup sizes, particularly in the female subgroup (*n* = 42), which limits statistical power and increasing the risk of residual confounding. Second, the MATEO trial was not powered or stratified for sex- or age-based comparisons. Accordingly, all findings should be considered hypothesis-generating.

## Conclusion

In this post hoc analysis of the MATEO trial exploring different maintenance strategies, in pts without disease progression after 3 months of platinum-based ctx, the distribution of primary tumor location differed according to sex, with female pts having more distal stomach tumors and male pts having more esophagogastric junction tumors. Female patients showed an improved PFS and OS in univariate analysis, though this was not a significant cofactor in multivariate analysis of OS. Similar survival outcomes were observed in younger versus older pts. In addition, toxicity did not differ clinically meaningful between sex and age subgroups. These findings support the growing evidence that sex is a relevant prognostic variable in EGA and that age alone should not preclude standard treatment approaches. Future prospective studies with adequate power and pre-specified sex- and age-stratified analyses are warranted to validate these findings and to elucidate the underlying biological mechanisms.

## Supplementary Information

Supplementary Material 1.

## Data Availability

The data that support the findings of this study are available from the corresponding author upon reasonable request.
