# Supplementary material for: Association of sex and age on outcomes of maintenance treatment in pati​ents with advanced esophagogastric adenocarcinoma: an exploratory analysis of the randomized AIO-YMO-0111-MATEO trial
Source: J Cancer Res Clin Oncol. 2026 Nov 13;152(11):228. doi: 10.1007/s00432-026-06662-y (PMC13768524; doi:10.1007/s00432-026-06662-y)
Supplement: Supplementary file 1 [file 432_2026_6662_MOESM1_ESM.docx]

| **Supplementary table 1** | | | | | | | |
| --- | --- | --- | --- | --- | --- | --- | --- |
| **Safety population**  **n= 155** | **Male patients**  **n= 113 (%)** | **Female patients**  **n= 42 (%)** | **p-value** |  | **<70y**  **n= 96 (%)** | **≥70y**  **n= 59 (%)** | **p-value** |
| **Leukopenie** | **4 (3.5)** | **1 (2.4)** | >0.9999 |  | **3 (3.1)** | **2 (3.4)** | >0.9999 |
| - CTC AE 1° | 3 (2.7) | 1 (2.4) |  |  | 2 (2.1) | 2 (3.4) |  |
| - CTC AE 2° | 1 (0.9) | - |  |  | 1 (1.0) | - |  |
| - CTC AE 3° | **-** | - |  |  | - | - |  |
| - CTC AE 4° | **-** | - |  |  | - | - |  |
| **Neutropenia** | **2 (1.8)** | **1 (2.4)** | >0.9999 |  | **3 (3.1)** | **-** | 0.2881 |
| - CTC AE 1° | **-** | - |  |  | - | - |  |
| - CTC AE 2° | 1 (0.9) | 1 (2.4) |  |  | 2 (2.1) | - |  |
| - CTC AE 3° | 1 (0.9) | - |  |  | 1 (1.0) | - |  |
| - CTC AE 4° | - | - |  |  | - | - |  |
| **Thrombocytopenia** | **5 (4.4)** | **2 (4.8)** | >0.9999 |  | **2 (2.1)** | **5 (8.5)** | 0.1060 |
| - CTC AE 1° | 3 (2.7) | 2 (4.8) |  |  | 1 (1.0) | 4 (6.8) |  |
| - CTC AE 2° | - | - |  |  | - | - |  |
| - CTC AE 3° | 2 (1.8) |  |  |  | 1 (1.0) | 1 (1.7) |  |
| - CTC AE 4° | **-** | - |  |  | - | - |  |
| **Anaemia** | **5 (4.4)** | **5 (11.9)** | 0.1356 |  | **6 (6.3)** | **4 (6.8)** | >0.9999 |
| - CTC AE 1° | 2 (1.8) | 4 (9.5) |  |  | 3 (3.1) | 3 (5.1) |  |
| - CTC AE 2° | 2 (1.8) | - |  |  | 1 (1.0) | 1 (1.7) |  |
| - CTC AE 3° | 1 (0.9) | 1 (2.4) |  |  | 2 (2.1) | - |  |
| - CTC AE 4° | - |  |  |  | **-** | **-** |  |
| **Diarrhea** | **24 (21.2)** | **8 (19.0)** | 0.8271 |  | **24 (25.0)** | **8 (13.6)** | 0.1040 |
| - CTC AE 1° | 13 (11.5) | 4 (9.5) |  |  | 14 (14.6) | 3 (5.1) |  |
| - CTC AE 2° | 9 (8.0) | 2 (4.8) |  |  | 6 (6.3) | 5 (8.5) |  |
| - CTC AE 3° | 2 (1.8) | 2 (4.8) |  |  | 4 (4.2) | - |  |
| - CTC AE 4° | **-** | **-** |  |  | **-** | **-** |  |
| **Nausea** | **19 (16.8)** | **11 (26.2)** | 0.2518 |  | **22 (22.9)** | **8 (13.6)** | 0.2090 |
| - CTC AE 1° | 16 (14.2) | 8 (19.0) |  |  | 19 (19.8) | 5 (8.5) |  |
| - CTC AE 2° | 2 (1.8) | 2 (4.8) |  |  | 2 (2.1) | 2 (3.4) |  |
| - CTC AE 3° | 1 (0.9) | 1 (2.4) |  |  | 1 (1) | 1 (1.7) |  |
| - CTC AE 4° | **-** | **-** |  |  | **-** | **-** |  |
| **Stomatitis** | **10 (8.8)** | **4 (9.5)** | >0.9999 |  | **11 (11.5)** | **3 (5.1)** | 0.2514 |
| - CTC AE 1° | 5 (4.4) | 1 (2.4) |  |  | 6 (6.3) | 0 |  |
| - CTC AE 2° | 3 (2.7) | 1 (2.4) |  |  | 3 (3.1) | 1 (1.7) |  |
| - CTC AE 3° | 2 (1.8) | 2 (4.8) |  |  | 2 (2.1) | 2 (3.4) |  |
| - CTC AE 4° | **-** | **-** |  |  | **-** | **-** |  |
| **Vomiting** | **13 (11.5)** | **6 (14.2)** | 0.5949 |  | **12 (12.5)** | **7 (11.9)** | >0.9999 |
| - CTC AE 1° | 12 (10.6) | 5 (11.9) |  |  | 11 (11.5) | 6 (10.2) |  |
| - CTC AE 2° | - | 1 (2.4) |  |  | - | 1 (1.7) |  |
| - CTC AE 3° | 1 (0.9) | - |  |  | 1 (1.0) | - |  |
| - CTC AE 4° | - | - |  |  | **-** | **-** |  |
| **Fatigue** | **19 (16.8)** | **8 (19.0)** | 0.8125 |  | **16 (16.7)** | **11 (18.6)** | 0.8283 |
| - CTC AE 1° | 9 (8.0) | 5 (11.9) |  |  | 10 (10.4) | 4 (6.8) |  |
| - CTC AE 2° | 7 (6.2) | 1 (2.4) |  |  | 4 (4.2) | 4 (6.8) |  |
| - CTC AE 3° | 3 (2.7) | 2 (4.8) |  |  | 2 (2.1) | 3 (5.1) |  |
| - CTC AE 4° | **-** |  |  |  | - | - |  |
| **Peripheral Sensory Neuropathy** | **27 (23.9)** | **13 (31.0)** | 0.4113 |  | **24 (25.0)** | **16 (27.1)** | 0.8505 |
| - CTC AE 1° | 5 (4.4) | 7 (16.7) |  |  | 7 (7.3) | 5 (8.5) |  |
| - CTC AE 2° | 17 (15.0) | 5 (11.9) |  |  | 13 (13.5) | 9 (15.3) |  |
| - CTC AE 3° | 5 (4.4) | 1 (2.4) |  |  | 4 (4.2) | 2 (3.4) |  |
| - CTC AE 4° | - |  |  |  | - | - |  |
| **Polyneuropathy** | **7 (6.2)** | **2 (4.8)** | >0.9999 |  | **7 (7.3)** | **2 (3.4)** | 0.4840 |
| - CTC AE 1° | 1 (0.9) | 1 (2.4) |  |  | 2 (2.1) | 0 |  |
| - CTC AE 2° | 4 (3.5) | 1 (2.4) |  |  | 3 (3.1) | 2 (3.4) |  |
| - CTC AE 3° | 2 (1.8) | 0 |  |  | 2 (2.1) | 0 |  |
| - CTC AE 4° | - |  |  |  | - | - |  |
| **Palmar-Plantar Erythrodysaesthesia** | **11 (9.7)** | **4 (9.5)** | >0.9999 |  | **11 (11.5)** | **4 (6.8)** | 0.4114 |
| - CTC AE 1° | 7 (6.2) | 1 (2.4) |  |  | 6 (6.3) | 2 (3.4) |  |
| - CTC AE 2° | 3 (2.7) | 3 (7.1) |  |  | 4 (4.2) | 2 (3.4) |  |
| - CTC AE 3° | 1 (0.9) | - |  |  | 1 (1.0) | - |  |
| - CTC 4° | - | - |  |  | **-** | **-** |  |
| **Decreased appetite** | **18 (15.9)** | **4 (9.5)** | 0.4387 |  | **11 (11.5)** | **11 (18.6)** | 0.2408 |
| - CTC AE 1° | 13 (11.5) | 4 (9.5) |  |  | 9 (9.4) | 8 (13.6) |  |
| - CTC AE 2° | 4 (3.5) | - |  |  | 2 (2.1) | 2 (3.4) |  |
| - CTC AE 3° | 1 (0.9) | - |  |  | - | 1 (1.7) |  |
| - CTC AE 4° | - | - |  |  | **-** | **-** |  |
| **Eye Lacrimation increased** | **9 (8.0)** | **6 (14.3)** | 0.2366 |  | **12 (12.5)** | **3 (5.1)** | 0.1667 |
| - CTC AE 1° | 8 (7.1) | 4 (9.5) |  |  | 11 (11.5) | 1 (1.7) |  |
| - CTC AE 2° | 1 (0.9) | 2 (4.8) |  |  | 1 (1.0) | 2 (3.4) |  |
| - CTC AE 3° | - | - |  |  | - | -- |  |
| - CTC AE 4° | - | - |  |  | - | - |  |
| **Abdominal pain** | **3 (2.7)** | **4 (9.5)** | 0.0867 |  | **5 (5.2)** | **2 (3.4)** | 0.7095 |
| - CTC AE 1° | 1 (0.9) | 4 (9.5) |  |  | 4 (4.2) | 2 (3.4) |  |
| - CTC AE 2° | 2 (1.8) | - |  |  | 1 (1.0) | - |  |
| - CTC AE 3° | - | - |  |  | - | - |  |
| - CTC AE 4° | - | - |  |  | - | - |  |
| **Constipation** | **5 (4.4)** | **3 (7.1)** | 0.4478 |  | **5 (5.2)** | **3 (5.1)** | >0.9999 |
| - CTC AE 1° | 3 (2.7) | 2 (4.8) |  |  | 3 (3.1) | 2 (3.4) |  |
| - CTC AE 2° | 2 (1.8) | 1 (2.4) |  |  | 2 (2.1) | 1 (1.7) |  |
| - CTC AE 3° | - | - |  |  | - | - |  |
| - CTC AE 4° | - | - |  |  | - | - |  |
| **Edema peripheral** | **3 (2.7)** | **3 (7.1)** | 0.3450 |  | **3 (3.1)** | **3 (5.1)** | 0.6745 |
| - CTC AE 1° | 1 (0.9) | - |  |  | 1 (1.0) | - |  |
| - CTC AE 2° | 2 (1.8) | 3 (7.1) |  |  | 2 (2.1) | 3 (5.1) |  |
| - CTC AE 3° | - | - |  |  | - | - |  |
| - CTC AE 4° | - | - |  |  | - | - |  |
| **Conjunctivitis** | **5 (4.4)** | **1 (2.4)** | >0.9999 |  | **4 (4.2)** | **2 (3.4)** | >0.9999 |
| - CTC AE 1° | 4 (3.5) | - |  |  | 2 (2.1) | 2 (3.4) |  |
| - CTC AE 2° | 1 (0.9) | 1 (2.4) |  |  | 2 (2.1) | - |  |
| - CTC AE 3° | - | - |  |  | - | - |  |
| - CTC AE 4° | - | - |  |  | - | - |  |
| **Blood bilirubin increased** | **3 (2.7)** | **2.4 (4.8)** | 0.6129 |  | **5 (5.2)** | **-** | 0.1572 |
| - CTC AE 1° | 1 (0.9) | - |  |  | 1 (1.0) | - |  |
| - CTC AE 2° | 2 (1.8) | 2 (4.8) |  |  | 4 (4.2) | - |  |
| - CTC AE 3° | - | - |  |  | - | - |  |
| - CTC AE 4° | - | - |  |  | - | - |  |
| **Neutrophil count decreased** | **6 (5.3)** | **-** | 0.1910 |  | **3 (3.1)** | **3 (5.1)** | 0.6745 |
| - CTC AE 1° | 2 (1.8) | - |  |  | - | 2 (3.4) |  |
| - CTC AE 2° | 3 (2.7) | - |  |  | 2 (2.1) | 1 (1.7) |  |
| - CTC AE 3° | - | - |  |  | - | - |  |
| - CTC AE 4° | 1 (0.9) | - |  |  | 1 (1.0) | - |  |
| **Platelet count decreased** | **5 (4.4)** | **1 (2.4)** | >0.9999 |  | **3 (3.1)** | **3 (5.1)** | 0.6745 |
| - CTC AE 1° | 2 (1.8) | 1 (2.4) |  |  | 1 (1.0) | 2 (3.4) |  |
| - CTC AE 2° | 2 (1.8) | - |  |  | 1 (1.0) | 1 (1.7) |  |
| - CTC AE 3° | 1 (0.9) | - |  |  | 1 (1.0) | - |  |
| - CTC AE 4° | - |  |  |  | - | **-** |  |
| **White blood cell count decreased** | **4 (3.5)** | **5 (11.9)** | 0.0613 |  | **8 (8.3)** | **1 (1.7)** | 0.1544 |
| - CTC AE 1° | 2 (1.8) | 2 (4.8) |  |  | 3 (3.1) | 1 (1.7) |  |
| - CTC AE 2° | 2 (1.8) | 2 (4.8) |  |  | 4 (4.2) | - |  |
| - CTC AE 3° | - | 1 (2.4) |  |  | 1 (1.0) | - |  |
| - CTC AE 4° | - | - |  |  | - | - |  |
| **Hypokalemia** | **5 (4.4)** | **2 (4.8)** | >0.9999 |  | **3 (3.1)** | **4 (6.8)** | 0.4281 |
| - CTC AE 1° | 3 (2.7) | 2 (4.8) |  |  | 2 (2.1) | 3 (5.1) |  |
| - CTC AE 2° | 1 (0.9) | - |  |  | - | 1 (1.7) |  |
| - CTC AE 3° | 1 (0.9) | - |  |  | 1 (1.0) | - |  |
| - CTC AE 4° |  | - |  |  | - | - |  |
| **Paraesthesia** | **2 (1.8)** | **1 (2.4)** | >0.9999 |  | **-** | **3 (5.1)** | 0.0534 |
| - CTC AE 1° | 1 (0.9) | - |  |  | - | 1 (1.7) |  |
| - CTC AE 2° | 1 (0.9) | 1 (2.4) |  |  | - | 2 (3.4) |  |
| - CTC AE 3° | - | - |  |  | - | - |  |
| - CTC AE 4° | - | - |  |  | - | - |  |
| **Alopecia** | **5 (4.4)** | **6 (14.3)** | 0.0704 |  | **9 (9.4)** | **2 (3.4)** | 0.2075 |
| - CTC AE 1° | 4 (3.5) | 3 (7.1) |  |  | 6 (6.3) | 1 (1.7) |  |
| - CTC AE 2° | 1 (0.9) | 1 (2.4) |  |  | 1 (1.0) | 1 (1.7) |  |
| - CTC AE 3° | - | 2 (4.8) |  |  | 2 (2.1) | - |  |
| - CTC AE 4° | - | - |  |  | - | - |  |
| **Erythema** | **3 (2.7)** | **3 (7.1)** | 0.3450 |  | **5 (5.2)** | **1 (1.7)** | 0.4085 |
| - CTC AE 1° | 2 (1.8) | 2 (4.8) |  |  | 4 (4.2) | - |  |
| - CTC AE 2° | 1 (0.9) | 1 (2.4) |  |  | 1 (1.0) | 1 (1.7) |  |
| - CTC AE 3° | - | - |  |  | - | - |  |
| - CTC AE 4° | - | - |  |  | - | - |  |
| **Pruritus** | **3 (2.7)** | **3 (7.1)** | 0.3450 |  | **4 (4.2)** | **2 (3.4)** | >0.9999 |
| - CTC AE 1° | 2 (1.8) | 3 (7.1) |  |  | 4 (4.2) | 1 (1.7) |  |
| - CTC AE 2° | 1 (0.9) | - |  |  | - | 1 (1.7) |  |
| - CTC AE 3° |  | - |  |  | - | - |  |
| - CTC AE 4° |  | - |  |  | - | - |  |
